# Severe caries are a clue for child neglect: a case report

**DOI:** 10.1186/s13256-018-1639-6

**Published:** 2018-04-26

**Authors:** Henk Sillevis Smitt, Nancy Mintjes, Reinanke Hovens, Jenny de Leeuw, Tjalling de Vries

**Affiliations:** 1 Department of Orofacial Surgery, Nij Smellinghe, Drachten, The Netherlands; 2 Department of Paediatrics, Medical Centre Leeuwarden, P.O. Box 888, 8901 BR Leeuwarden, The Netherlands; 3 Department of Special Care Dentistry, Medical Centre Leeuwarden, Leeuwarden, The Netherlands; 4 Public Health, Advice and Support Centre for Domestic Violence and Child Abuse (Veilig Thuis), Leeuwarden, The Netherlands

**Keywords:** Dental caries, Child abuse and neglect, Dental care, Parenting

## Abstract

**Background:**

Child abuse and neglect have strong negative effects on the well-being of children, not only during childhood but also later in life. Therefore, early recognition is important.

**Case presentation:**

We describe a 4-year-old Caucasian boy who had severe dental caries. This was a result of insufficient dental care: he refused to brush his teeth and drank sweetened drinks. We considered this dental neglect to be a manifestation of child neglect and social services were consequently called in to help the family.

**Conclusions:**

There is a strong association between child abuse and neglect and dental caries. Abused children often have severe dental caries and in children who had dental caries, child abuse and neglect is often established. An important factor is insufficient parenting; therefore, we believe that severe dental caries is an important indicator for child abuse and neglect.

## Background

Child abuse and neglect have a strongly negative effect on the developing child. These effects not only disturb the emotional, psychological, and physiological well-being of a child during childhood, but can also have detrimental effects later in life [1, 2]. Early recognition is therefore important. Recently, we encountered a child in whom severe dental caries led to identification of child neglect. In this article, we describe this child and discuss the relationship between dental caries and child abuse and neglect.

## Case presentation

A 4-year-old Caucasian boy was referred for multiple tooth extraction under general anesthesia. He had undergone this procedure earlier at the age of 3 years and at this time his parents had been instructed about dental hygiene, including regular brushing of teeth and avoiding sweetened drinks. His history was otherwise unremarkable; he had never been referred to an Emergency Room and had no history of bone fractures.

He weighed 14 kg, −2 standard deviation (sd); his length was measured at 95 cm (−2 sd). A physical examination was normal and there were no bruises or other signs of physical trauma.

When he returned from the operation theatre he asked for sweetened drinks in a bottle and, although this was discouraged by the pediatric nurse, his mother consoled him with a bottle containing a sweetened drink. His parents admitted that they found it difficult to handle him and rarely dared to deny him what he asked for. They also stated that when he refused to brush his teeth, they stopped their efforts to do so.

This was interpreted as dental neglect as a result of insufficient parenting. Moreover, we foresaw further future problems. These were discussed with the boy’s parents and we proposed to refer the family to social services. The parents agreed and intensive pedagogical help in parenting was offered. After 1 year, the child no longer had teeth affected by caries; in addition, he brushed his teeth twice daily and was doing well. His parents reported improvement in their parenting skills and social services confirmed this.

## Discussion

In this child, severe dental caries was the first symptom of child neglect. With the help of social services, the child and his family could receive adequate support and further mistreatment was prevented.

Child abuse includes many aspects of abuse: physical violence; sexual abuse; physical, emotional, or psychological neglect; emotional or psychological abuse; (witnessing) domestic violence; and factitious illness disorder. In most children, there is an overlap between different forms of child abuse.

Dental caries is a multifactorial disease caused by oral bacteria and influenced by: genetic, socio-economic, and dietary factors; fluoride exposure; and oral hygiene practices [3]. Mutans streptococci adhere to enamel and produce large amounts of acids, leading to enamel destruction; sugars further fuel this process. Fluoride, in contrast, protects against caries. Thus, the restriction of dietary sugar, brushing one’s teeth twice a day with toothpaste that contains fluoride, and regular check-ups by a dentist are important methods to protect the teeth from caries.

We believe that severe dental caries is an early symptom of child abuse and neglect and this is supported by the literature. In a group of 66 children with a history of child abuse and neglect, 38 (58%) had severe caries [4]. In a recent systematic review, nine studies could be included on the relationship between dental caries and neglect: all in all, representing 1595 children. Factors leading to dental neglect that were identified included failure to provide basic oral care, failure or delay in seeking dental treatment, and failure to comply with treatment [5].

In addition, we studied a group of 205 children who underwent multiple tooth extraction because of dental caries. In this group, the diagnosis of child abuse and neglect had been formally established within 9 years after the procedure for 47 of these children: 23%; 95% confidence interval (95% CI), 20–26%. In 27 children (13%; 95% CI, 11–15%), the procedure occurred before child abuse was established. We also found that the average interval from tooth extraction to reporting to social services was 36 months (6 to 91 months) [6].

Basic dental care includes regular tooth brushing and visiting a dentist twice yearly. Both require time, attention, care, and parenting skills. Parents of neglected children often lack these elements. In a case–control study, the relationship between lack of parenting skills and dental caries was demonstrated by comparing 28 children with dental caries with 26 healthy controls [7]. In this rather elegant study, parents and children were videotaped and their interactions were coded in a structured manner by trained observers who were blind to the dental condition. This study showed that controls had significantly higher scores compared to cases on the dimensions of positive involvement, encouragement, problem solving, and interpersonal atmosphere. The parents of controls were also less likely to show coercive behaviors.

Likewise, a study of 114 children in Estonia who underwent dental extraction under anesthesia due to severe caries revealed that, although the score of quality of life had improved 6 months after the procedure, the process that led to the caries still continued [8]. This indicates that undergoing the procedure itself does not necessarily affect dental care by the parents.

## Conclusions

Insufficient dental care by parents can be seen as dental neglect and therefore is a sign of child neglect. Health care workers involved in the care of children are thus in a unique position to recognize early symptoms of child abuse and neglect. We believe that the presence of dental caries is an important clue to this diagnosis. Anyone involved in the care of children should therefore be aware that dental caries is an early sign of child neglect. For this reason, families with children with severe dental caries should be offered help and support that is not restricted to dental care but also encompasses social services.

## References

[1] Felitti VJ, Anda RF, Nordenberg D, *et al*. Relationship of childhood abuse and household dysfunction to many of the leading causes of death in adults. Am J Prev Med. 1998;14:245–58. 10.1016/s0749-3797(98)00017-8 9635069

[2] Gilbert LK, Breiding MJ, Merrick MT, *et al*. Childhood adversity and adult chronic disease: an update from ten states and the District of Columbia, 2010. Am J Prev Med. 2015;48:345–9. 10.1016/j.amepre.2014.09.006 25300735

[3] Ng MW, Chase I. Early childhood caries: risk-based disease prevention and management. Dent Clin N Am. 2013;57:1–16. 10.1016/j.cden.2012.09.002 23174607

[4] Valencia-Rojas N, Lawrence HP, Goodman D. Prevalence of early childhood caries in a population of children with history of maltreatment. J Public Health Dent. 2008;68:94–101. 10.1111/j.1752-7325.2007.00077.x 18248339

[5] Bhatia SK, Maguire SA, Chadwick BL, Hunter ML, Harris JC, Tempest V, Mann MK, Kemp AM. Characteristics of child dental neglect: a systematic review. J Dent. 2014;42:229–39. 10.1016/j.jdent.2013.10.010 24140926

[6] Sillevis Smitt H, de Leeuw J, de Vries T. Association between severe dental caries and child abuse and neglect. J Oral Maxillofac Surg. 2017. 10.1016/j.joms.2017.05.004. [Epub ahead of print]. 28586637

[7] de Jong-Lenters M, Duijster D, Bruist MA, Thijssen J, de Ruiter C. The relationship between parenting, family interaction and childhood dental caries: a case-control study. Soc Sci Med. 2014;116:49–55. 10.1016/j.socscimed.2014.06.031 24980791

[8] Jankauskiené B, Virtanen JI, Narbutaité J. Follow-up of children’s oral health-related quality of life after dental general anaesthesia treatment. Acta Odontol Scand. 2017;75:255–61. 10.1080/00016357.2017.1286034 28358287

